# Supplementary material for: Enhancing the Dye-Rejection Efficiencies and Stability of Graphene Oxide-Based Nanofiltration Membranes via Divalent Cation Intercalation and Mild Reduction
Source: Membranes (Basel). 2022 Apr 2;12(4):402. doi: 10.3390/membranes12040402 (PMC9031111; doi:10.3390/membranes12040402)
Supplement: Supplementary file 1 [file membranes-12-00402-s001.zip › membranes-1650404-supplementary.pdf]

Supplementary Material for

Dye Rejection Efficiencies and Stability Enhancement of Graphene Oxide-Based Nanofiltration Membranes via Divalent Cation Intercalation and Mild Reduction

Hobin Jee [1,†], Jaewon Jang [2,†], Yesol Kang [3], Tasnim Eisa [4], Kyu-Jung Chae [4], In S. Kim [3] and Euntae Yang [1,*]

1 Department of Marine Environmental Engineering, Gyeongsang National University, Tongyoung 53064, Korea; hbj99@gnu.ac.kr

2 KEPCO Research Institute (KEPRI), Korea Electric Power Corporation (KEPCO), Naju 58277, Korea; jwjang@gm.gist.ac.kr

3 School of Earth Sciences and Environmental Engineering, Gwangju Institute of Science and Technology (GIST), Gwangju 61005, Korea; yesol7964@gist.ac.kr (Y.K.); iskim@gist.ac.kr (I.S.K.)

4 Department of Environmental Engineering, Korea Maritime and Ocean University, Busan 49112, Korea; tasnim.i.eisa@gmail.com (T.E.); kkjdream@kmou.ac.kr (K.-J.C.)

* Correspondence: yet83@gnu.ac.kr

† These authors contributed equally to this work.

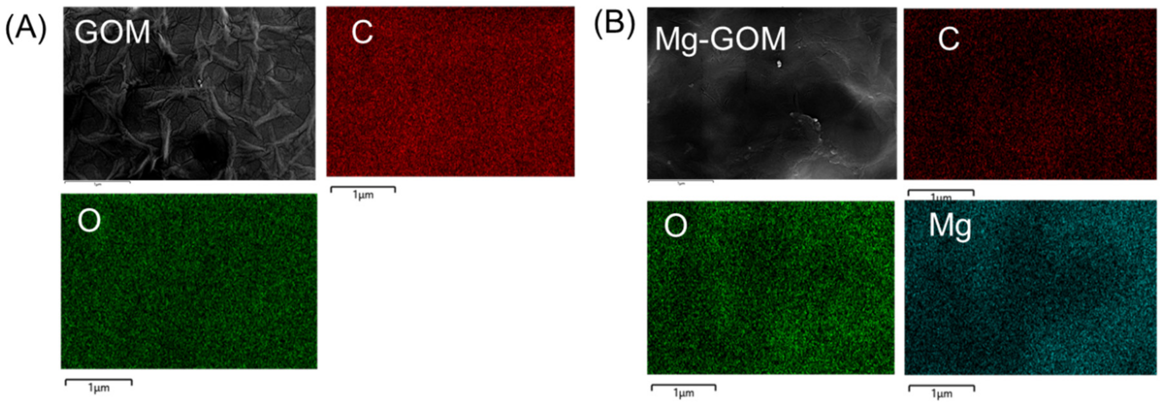

Figure S1. EDX mapping of (**A**) GOM and (**B**) Mg-GOM.

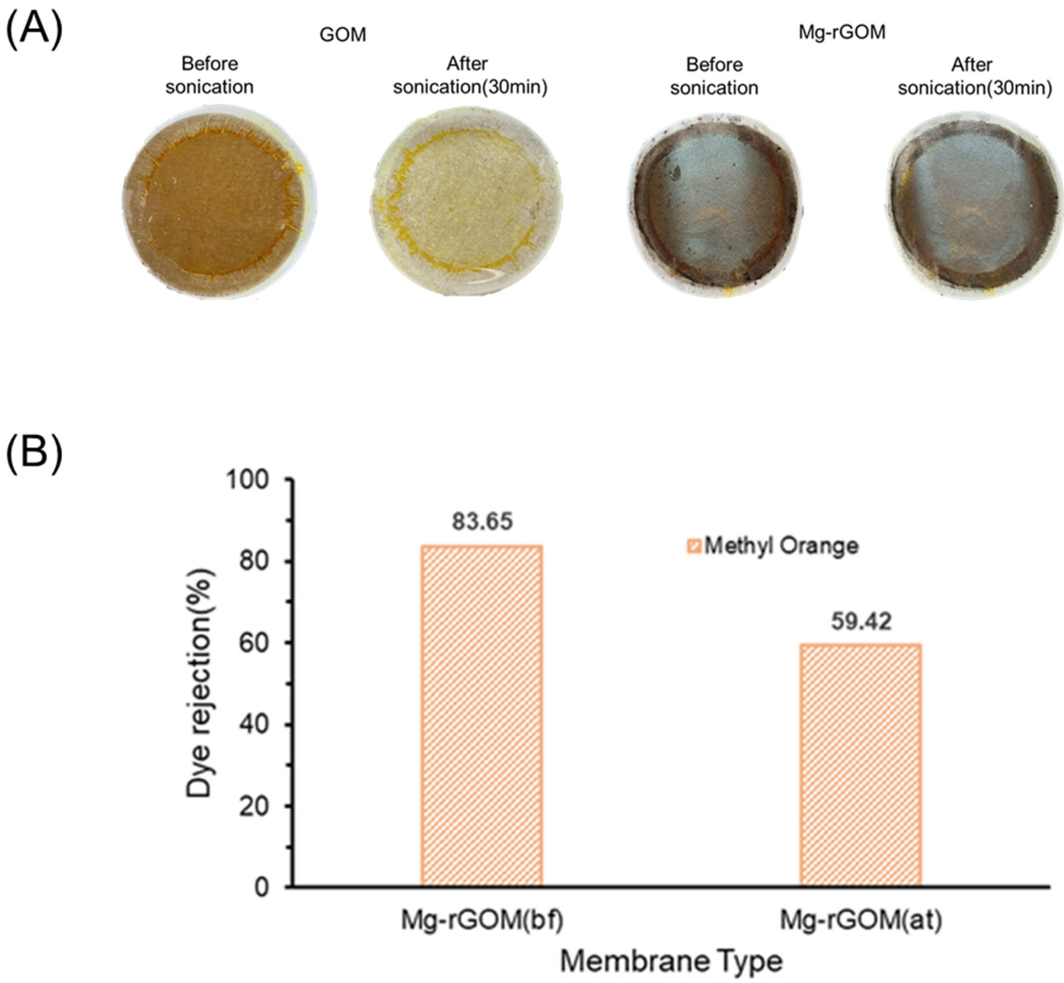

Figure S2. (**A**) Photographs of the laminar GO layers after rejection test with a methyl orange dye solution before and after 30-min sonication, and (**B**) comparison of methyl orange rejection efficiency of the Mg-rGOM(TH) before and after 30-min sonication.
